# Supplementary material for: Planar cell polarity proteins mediate ketamine-induced restoration of glutamatergic synapses in prefrontal cortical neurons in a mouse model for chronic stress
Source: Nat Commun. 2024 Jun 10;15:4945. doi: 10.1038/s41467-024-48257-6 (PMC11165002; doi:10.1038/s41467-024-48257-6)
Supplement: Supplementary file 1 — Supplementary Information [file 41467_2024_48257_MOESM1_ESM.pdf]

Supplemental Fig. 1. Cell Type Determination determined by Seurat FindAllMarkers function using default parameters fed into SCpubr package do_GroupwiseDEPlot function. Markers used to determine cell type for each cluster (1-18) are plotted in 3 different heatmaps (from top to bottom): statistical significance, fold change of average expression, average expression values.

Supplemental Fig. 2. CellChat and GO analyses. a. CellChat analysis of FGF Pathway using the ChordDiagram function. **b.** Top 10 pathway in GO analysis of pathways that underwent changes after corticosterone administration. **c.** Top 10 pathway in GO analysis of pathways that underwent changes after ketamine treatment of corticosterone administrated animals. **d.** The MAP kinase pathway after corticosterone administration. **e.** The MAP kinase pathway after ketamine treatment of corticosterone administrated animals.

Supplemental Fig. 3. Ligand-receptor pairs of ncWNT pathway added to CellChat. Cell-cell interactions added to CellChat database locally to include the ncWNT pathway in CellChat analysis.

Supplemental Fig. 4. Single cell sequencing data for PCP gene expression. a, schematics of experimental design. The illustrations were created with BioRender. **b,** Violin plots showing the changes of Celsr2, Celsr3 and Prickle2 expression in different cell types following treatment of mice with CORT for 6 weeks. **c,** Violin plots showing the changes of Celsr2, Celsr3 and Prickle2 expression in different cell types 24h after Ket treatment of animals which have been treated with CORT for 6 weeks.

Supplemental Fig. 5. Quality control for RNAscope Multiplex Fluorescent Assay. a, Schematic illustration of the experimental design. The illustrations were created with BioRender. **b**, Micrographs for RNAscope 3-plex negative control probes Dapb (Dihydrodipicolinate reductase of *Bacillus subtilis* strain). **c**, Micrographs for RNAscope 3-plex positive control probes Ppib (Peptidylprolyl Isomerase B), Polra (RNA Polymerase II Subunit A) and Ubc (Ubiquitin C). Scale bar 10 µm; Magnification ×40.

Supplemental Fig. 6. Confirmation of CRISPR-mediated knockout of Celsrs and Prickle2. a, Schematics of experimental design. The illustrations were created with BioRender. **b,** Immunostaining of Celsrs and Prickle. **c,** Quantification of b.

Supplemental Fig. 7. Effects of knocking out Celsr23 and PK2 in IL-PFC neurons projecting to the BLA on behavior and synapses. a, Schematics of experimental design. The illustrations were created with BioRender. **b,** Representative heatmaps for immobility time. **c,** Immobility time in the TST [F (2, 9) = 0.086, P = 0.9180]. **d,** Representative heatmaps for open field. **e,** Locomotion in the open field [F (2, 9) = 0.714, P = 0.5152]. **f,** Food consumed in the Food Consumption Test [F (2, 9) = 0.1803, P = 0.8379]. **g,** Representative micrographs. **h,** Quantification of dendritic spines [F (2, 6) = 5.796, P < 0.05]. **i,** Quantification of excitatory synapses [F (2, 6) = 17.31, P < 0.01]. Each column represents the mean + S.E.M. of 3-4 animals (7-14 neurons/animal). Statistical analysis was performed by One-way ANOVA followed by Tukey's test. *p<0.05

compared with the control group (Control sgRNA). Scale bar 3 µm; Magnification ×63. blue arrowhead, Basson puncta; red arrowhead, PSD95 puncta; white arrowhead, Bassoon/PSD95 puncta.

Supplemental Fig. 8. Sucrose preference test and body weight after Food Consumption Test a, Sucrose Preference Test (SPT). Chronic CORT treatment caused no significant effect on SPT as compared to Control-treated group [T=0.9102, P= 0.4142]. Each column represents the mean + S.E.M. of 3 animals. Statistical analysis was performed by Student's t-test. **b,** Body weight before and after Food Consumption Test. Mice food deprivation for Food Consumption Test caused a reduction of less than 10% of mouse body weight. Each column represents the mean of 17 animals before and after food deprivation.

Supplemental Fig. 9. Lower magnification images of excitatory synapses and dendritic spines. Representative micrographs illustrating excitatory synapses and dendritic spines labelling. Scale bar 5 µm; Magnification ×63. green, GFP; blue, Basson puncta; red, PSD95 puncta.

Supplementary Movie. S1. Three-Dimensional view of dendritic spines of a BLA-projecting IL PFC neuron. Neuron and dendritic spines labeled with GFP**.** Magnification ×63. green, GFP.

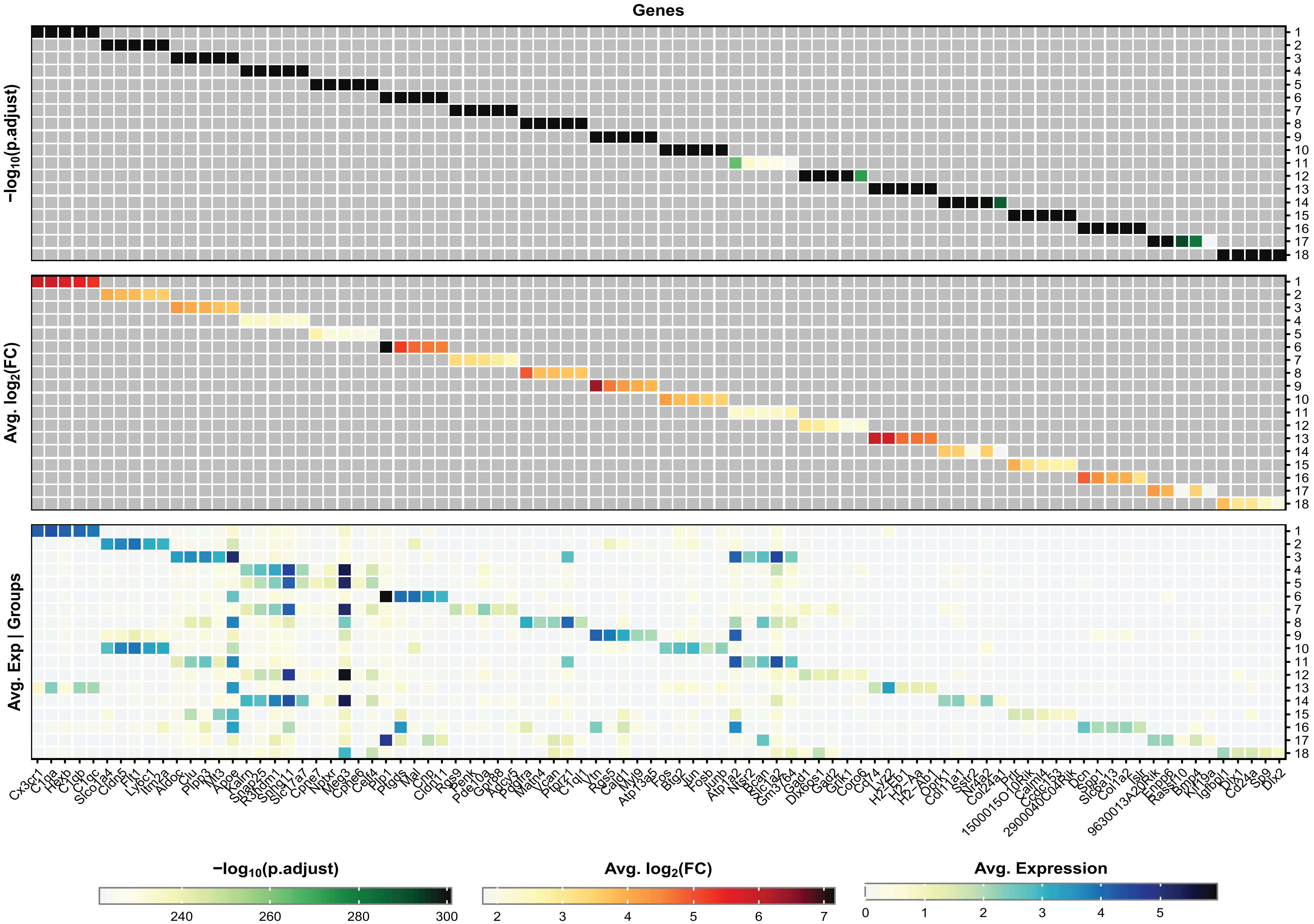

Genes
−log10(p.adjust)
Avg. log2(FC)
Avg. Exp | Groups
1
2
3
4
5
6
7
8
9
10
11
12
13
14
15
16
17
18
Cx3cr1
C1qa
Hexb
C1qb
C1qc
Slco1a4
Cldn5
Flt1
Ly6c1
Itm2a
Aldoc
Clu
Plpp3
Mt3
Apoe
Kalrn
Snap25
R3hdm1
Snhg11
Slc17a7
Cpne7
Nptxr
Meg3
Cpne6
Celf4
Plp1
Ptgds
Mal
Cnp
Cldn11
Rgs9
Penk
Pde10a
Gpr88
Adcy5
Pdgfra
Matn4
Vcan
Ptprz1
C1ql1
Vtn
Rgs5
Cald1
Myl9
Atp13a5
Fos
Btg2
Jun
Fosb
Junb
Atp1a2
Ntsr2
Bcan
Slc1a2
Gm3764
Gad1
Dlx6os1
Gad2
Grik1
Coro6
Cd74
Lyz2
H2-Eb1
H2-Aa
H2-Ab1
Oprk1
Col11a1
Sstr2
Nr4a2
Col24a1
Prlr
1500015O10Rik
Calml4
Ccdc153
2900040C04Rik
Dcn
Spp1
Slc6a13
Col1a2
Islr
9630013A20Rik
Enpp6
Rassf10
Bmp4
Kif19a
Igfbpl1
Dlx1
Cd24a
Sp9
Dlx2
−log10(p.adjust)
240
260
280
300
Avg. log2(FC)
2
3
4
5
6
7
Avg. Expression
0
1
2
3
4
5

a

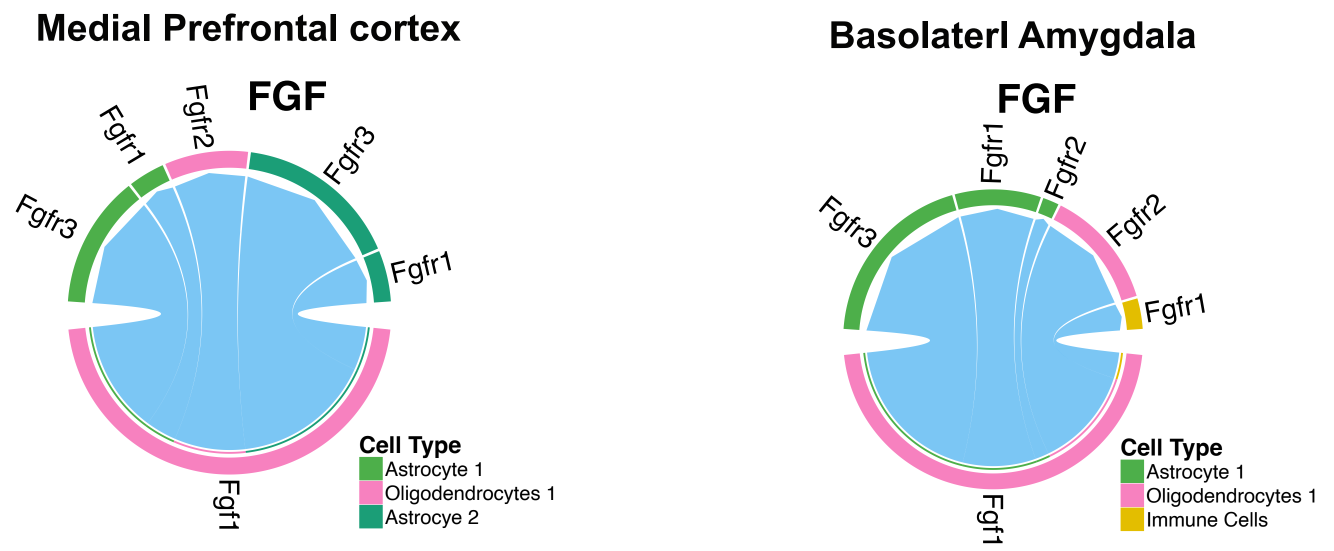

b

Corticosterone Administration

Pathway Name	P-Value
Neuroactive ligand-receptor interaction	5.10E-22
Metabolic pathways	7.17E-21
Pathways in cancer	3.77E-16
Calcium signaling pathway	4.27E-09
MAPK signaling pathway	2.07E-08
Breast cancer	3.95E-08
Signaling pathways regulating pluripotency of stem cells	1.04E-07
Hepatocellular carcinoma	2.18E-07
Focal adhesion	2.46E-07
Rap1 signaling pathway	5.24E-07

c

Ketamine treatment after Corticosterone Administration

Pathway Name	P-Value
Neuroactive ligand-receptor interaction	8.89E-24
Metabolic pathways	3.54E-14
Calcium signaling pathway	2.27E-09
Pathways in cancer	4.60E-08
Cushing syndrome	7.50E-06
cAMP signaling pathway	1.46E-05
Parathyroid hormone synthesis, secretion and action	2.65E-05
Axon guidance	3.39E-05
Protein digestion and absorption	4.46E-05
MAPK signaling pathway	8.81E-05

d

Corticosterone Administration

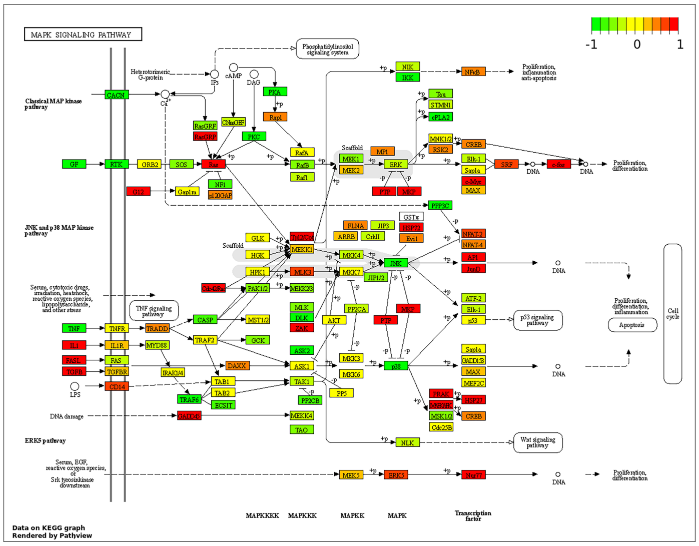

e

Ketamine treatment after Corticosterone Administration

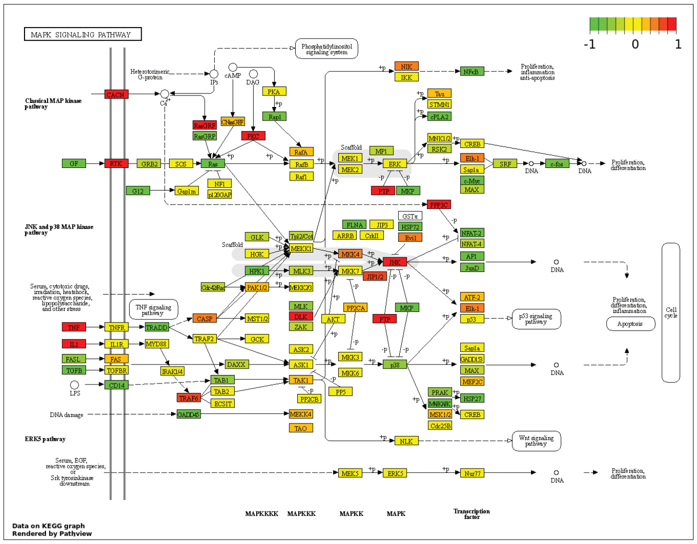

ncWNT Pathway Interactions

Interaction Name	Pathway Name	Ligand	Receptor	Annotation	Interaction Name 2
WNT4_FZD3	ncWNT	Wnt4	Fzd3	Secreted Signaling	Wnt4 - Fzd3
WNT4_FZD6	ncWNT	Wnt4	Fzd6	Secreted Signaling	Wnt4 - Fzd6
WNT5a_FZD6	ncWNT	Wnt5a	Fzd6	Secreted Signaling	Wnt5a - Fzd6
WNT5a_FZD6	ncWNT	Wnt5a	Fzd6	Secreted Signaling	Wnt5a - Fzd6
WNT7b_FZD6	ncWNT	Wnt7b	Fzd6	Secreted Signaling	Wnt7b - Fzd6
CELSR3_CELSR3	ncWNT	Celsr3	Celsr3	Cell-Cell Contact	Celsr3 - Celsr3
CELSR2_CELSR2	ncWNT	Celsr2	Celsr2	Cell-Cell Contact	Celsr2 - Celsr2
VANGL2_FZD3	ncWNT	Vangl2	Fzd3	Cell-Cell Contact	Vangl2 - Fzd3
FZD3_VANGL2	ncWNT	Fzd3	Vangl2	Cell-Cell Contact	Fzd3 - Vangl2

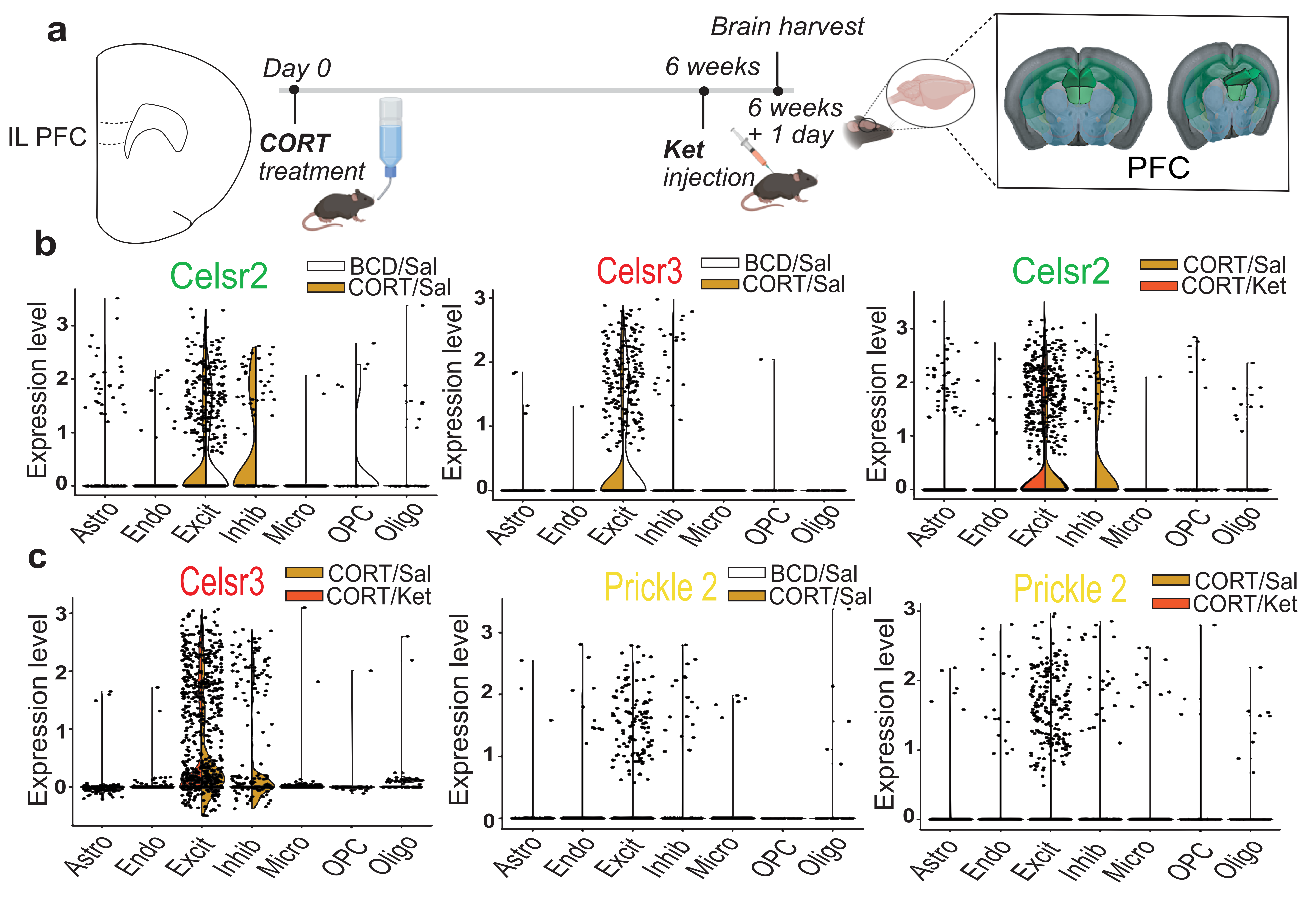

a
IL PFC
Day 0
CORT
treatment
6 weeks
Ket
injection
Brain harvest
6 weeks
+ 1 day
PFC
b
Celsr2
BCD/Sal
CORT/Sal
Expression level
Astro
Endo
Excit
Inhib
Micro
OPC
Oligo
Celsr3
BCD/Sal
CORT/Sal
Celsr2
CORT/Sal
CORT/Ket
c
Celsr3
CORT/Sal
CORT/Ket
Prickle 2
BCD/Sal
CORT/Sal
Prickle 2
CORT/Sal
CORT/Ket

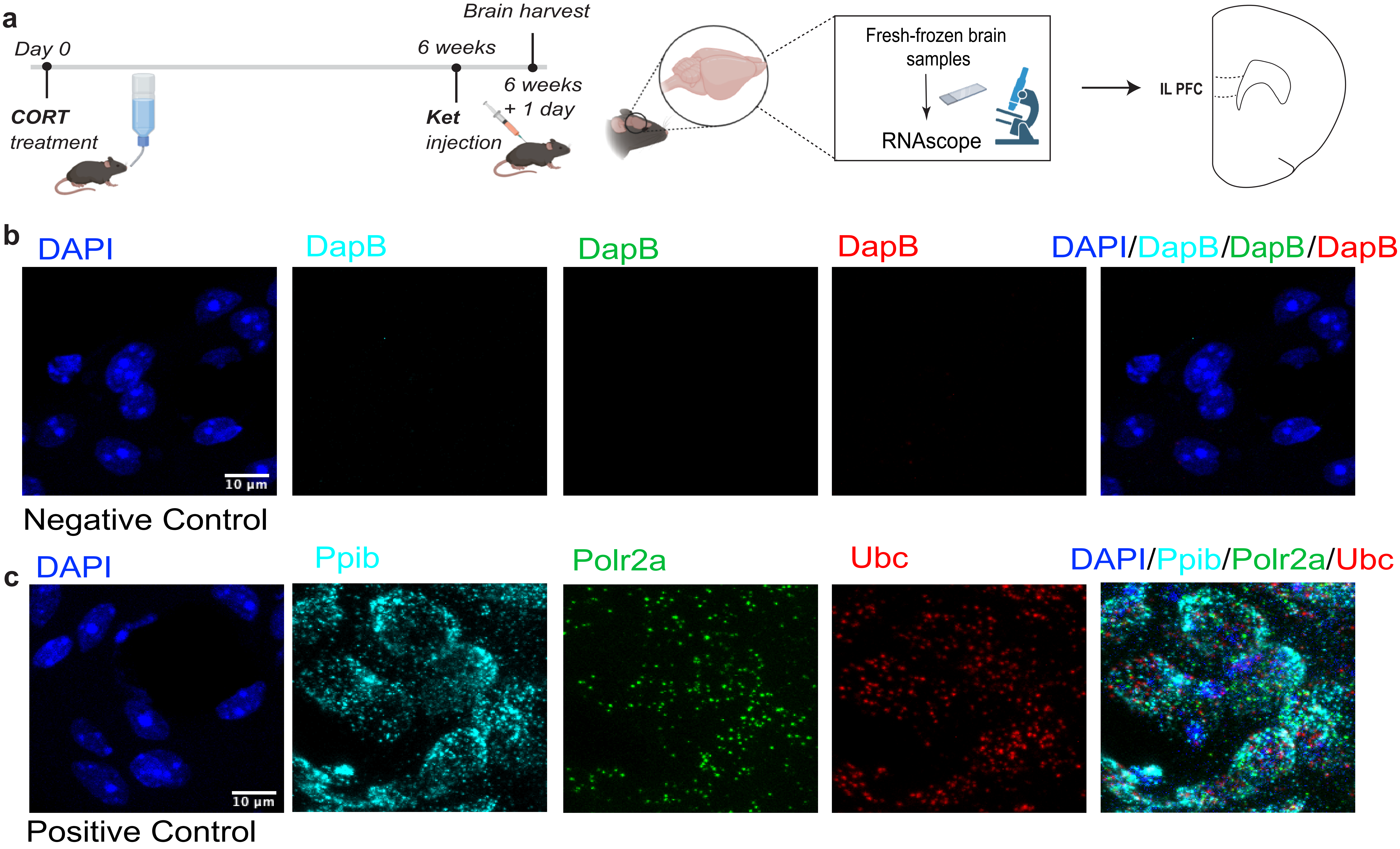
a
Day 0
CORT
treatment
6 weeks
Ket
injection
Brain harvest
6 weeks
+ 1 day
Fresh-frozen brain
samples
RNAscope
IL PFC
b
DAPI
DapB
DapB
DapB
DAPI/DapB/DapB/DapB
10 µm
Negative Control
c
DAPI
Ppib
Polr2a
Ubc
DAPI/Ppib/Polr2a/Ubc
10 µm
Positive Control

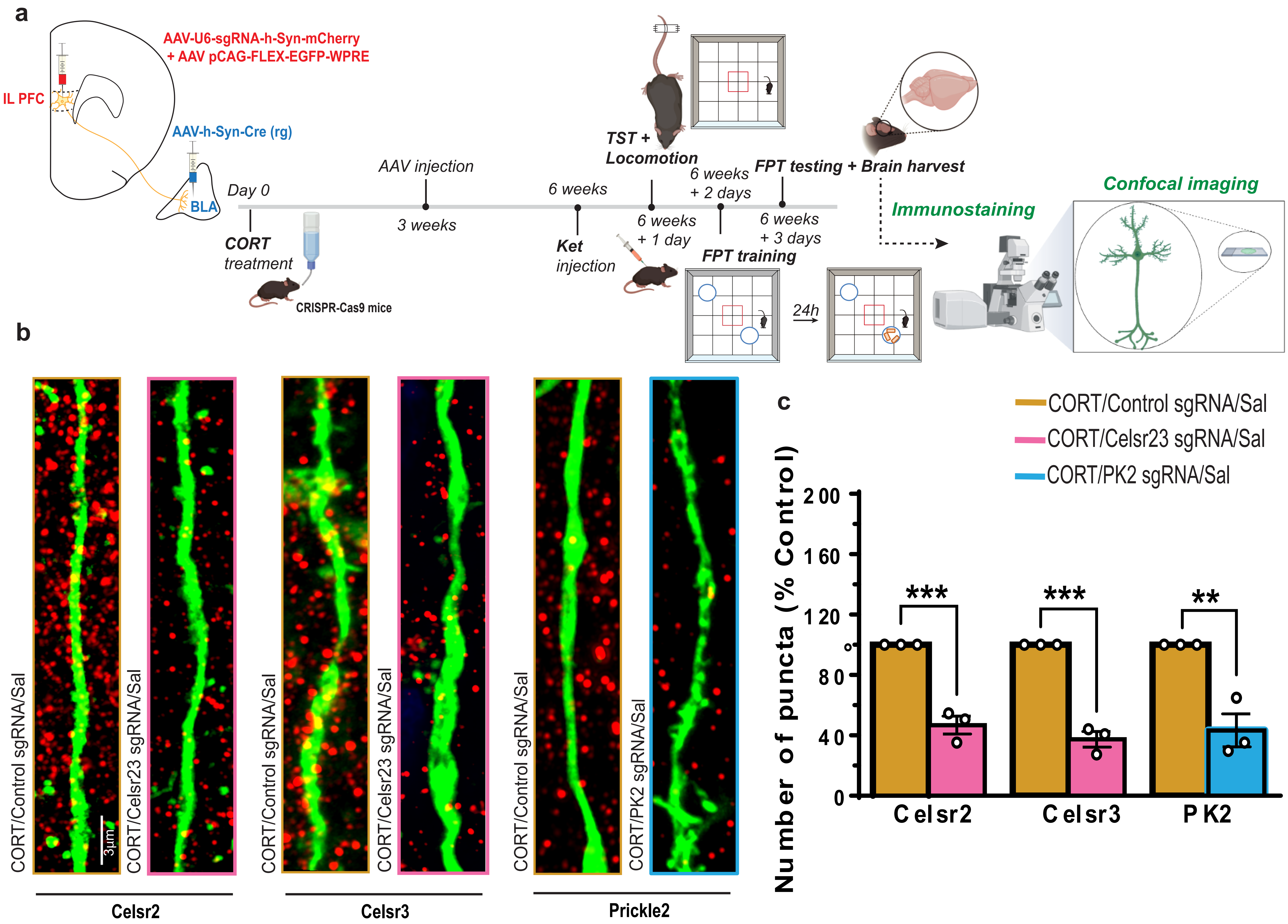
a
AAV-U6-sgRNA-h-Syn-mCherry
+ AAV pCAG-FLEX-EGFP-WPRE
IL PFC
AAV-h-Syn-Cre (rg)
BLA
Day 0
CORT
treatment
CRISPR-Cas9 mice
AAV injection
3 weeks
6 weeks
Ket
injection
TST +
Locomotion
6 weeks
+ 1 day
6 weeks
+ 2 days
FPT training
24h
FPT testing + Brain harvest
6 weeks
+ 3 days
Immunostaining
Confocal imaging
b
CORT/Control sgRNA/Sal
3µm
CORT/Celsr23 sgRNA/Sal
Celsr2
CORT/Control sgRNA/Sal
CORT/Celsr23 sgRNA/Sal
Celsr3
CORT/Control sgRNA/Sal
CORT/PK2 sgRNA/Sal
Prickle2
c
CORT/Control sgRNA/Sal
CORT/Celsr23 sgRNA/Sal
CORT/PK2 sgRNA/Sal
Number of puncta (% Control)
200
160
120
80
40
0
***
***
**
Celsr2
Celsr3
PK2

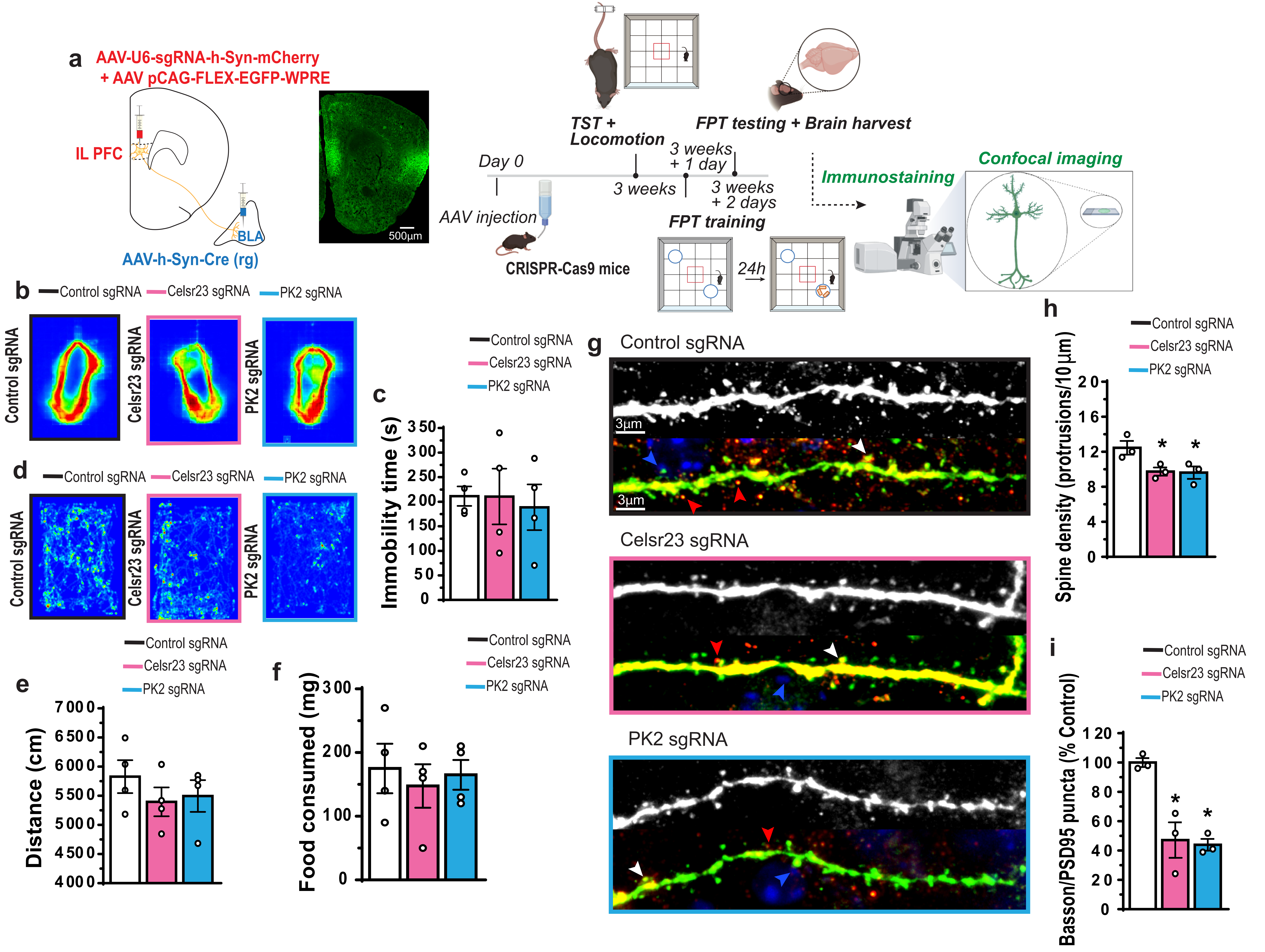
a
AAV-U6-sgRNA-h-Syn-mCherry
+ AAV pCAG-FLEX-EGFP-WPRE
IL PFC
BLA
AAV-h-Syn-Cre (rg)
500µm
TST + Locomotion
FPT testing + Brain harvest
Day 0
3 weeks
3 weeks + 1 day
3 weeks + 2 days
AAV injection
CRISPR-Cas9 mice
FPT training
24h
Immunostaining
Confocal imaging
b
Control sgRNA
Celsr23 sgRNA
PK2 sgRNA
c
Immobility time (s)
d
e
Distance (cm)
f
Food consumed (mg)
g
3µm
h
Spine density (protrusions/10 µm)
i
Basson/PSD95 puncta (% Control)

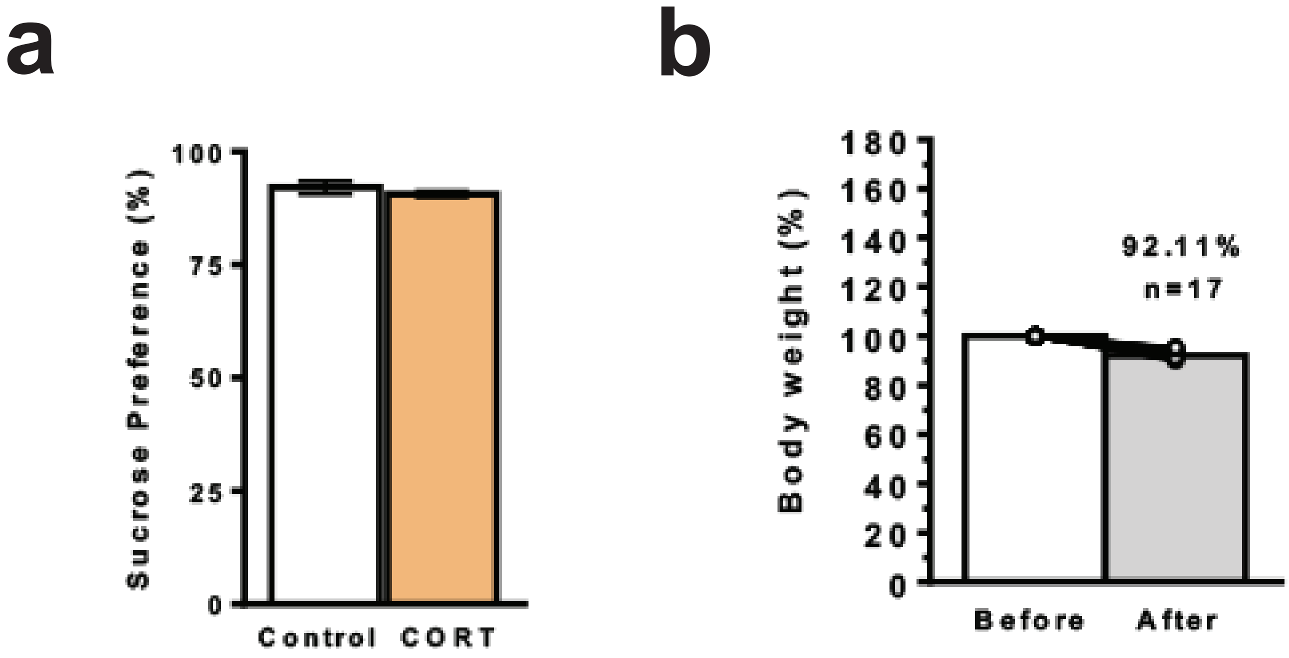
a
Sucrose Preference (%)
100
75
50
25
0
Control
CORT
b
Body weight (%)
180
160
140
120
100
80
60
40
20
0
92.11%
n=17
Before
After

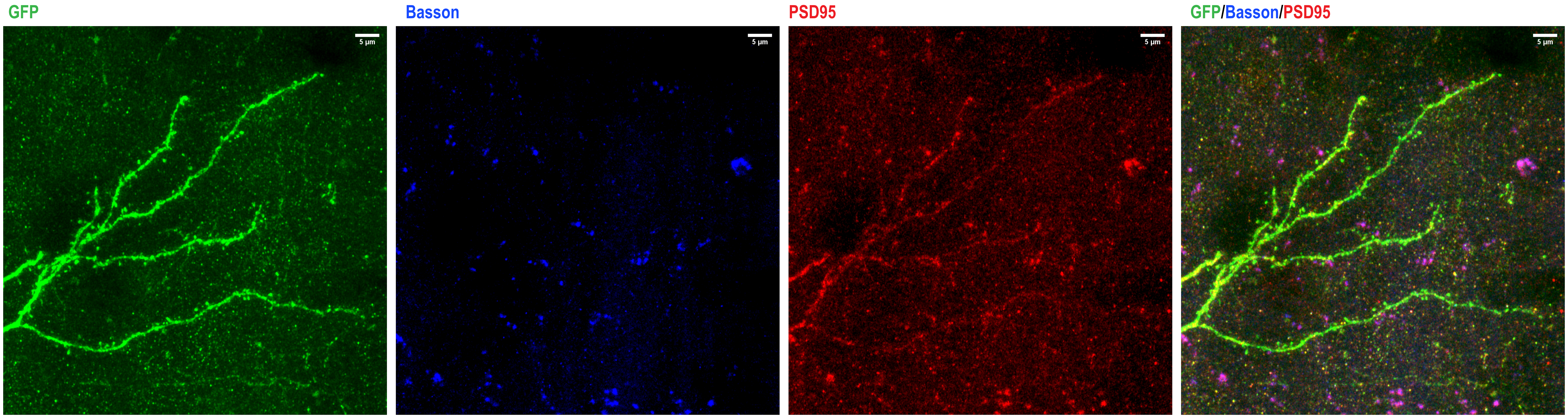
GFP
Basson
PSD95
GFP/Basson/PSD95
5 µm
5 µm
5 µm
5 µm
