# Supplementary material for: Planar cell polarity proteins mediate ketamine-induced restoration of glutamatergic synapses in prefrontal cortical neurons in a mouse model for chronic stress
Source: Nat Commun. 2024 Jun 10;15:4945. doi: 10.1038/s41467-024-48257-6 (PMC11165002; doi:10.1038/s41467-024-48257-6)
Supplement: Supplementary file 3 — Description of Additional Supplementary Information [file 41467_2024_48257_MOESM3_ESM.pdf]

Description of Additional Supplementary Information

File Name: Supplementary Movie. S1.

Description: Three-Dimensional view of dendritic spines of a BLA-projecting

731 IL PFC neuron. Neuron and dendritic spines labeled with GFP. Magnification ×63. green, GFP.
