# Supplementary material for: Planar cell polarity proteins mediate ketamine-induced restoration of glutamatergic synapses in prefrontal cortical neurons in a mouse model for chronic stress
Source: Nat Commun. 2024 Jun 10;15:4945. doi: 10.1038/s41467-024-48257-6 (PMC11165002; doi:10.1038/s41467-024-48257-6)
Supplement: Supplementary file 5 — Reporting Summary [file 41467_2024_48257_MOESM5_ESM.pdf]

nature portfolio

Corresponding author(s): Yimin Zou

Last updated by author(s): Mar 24, 2023

Reporting Summary

Nature Portfolio wishes to improve the reproducibility of the work that we publish. This form provides structure for consistency and transparency in reporting. For further information on Nature Portfolio policies, see our Editorial Policies and the Editorial Policy Checklist.

Statistics

For all statistical analyses, confirm that the following items are present in the figure legend, table legend, main text, or Methods section.

n/a	Confirmed	
☐	☒	The exact sample size (*n*) for each experimental group/condition, given as a discrete number and unit of measurement
☐	☒	A statement on whether measurements were taken from distinct samples or whether the same sample was measured repeatedly
☐	☒	The statistical test(s) used AND whether they are one- or two-sided<br>*Only common tests should be described solely by name; describe more complex techniques in the Methods section.*
☒	☐	A description of all covariates tested
☐	☒	A description of any assumptions or corrections, such as tests of normality and adjustment for multiple comparisons
☐	☒	A full description of the statistical parameters including central tendency (e.g. means) or other basic estimates (e.g. regression coefficient) AND variation (e.g. standard deviation) or associated estimates of uncertainty (e.g. confidence intervals)
☐	☒	For null hypothesis testing, the test statistic (e.g. *F*, *t*, *r*) with confidence intervals, effect sizes, degrees of freedom and *P* value noted<br>*Give P values as exact values whenever suitable.*
☒	☐	For Bayesian analysis, information on the choice of priors and Markov chain Monte Carlo settings
☒	☐	For hierarchical and complex designs, identification of the appropriate level for tests and full reporting of outcomes
☒	☐	Estimates of effect sizes (e.g. Cohen's *d*, Pearson's *r*), indicating how they were calculated

Our web collection on statistics for biologists contains articles on many of the points above.

Software and code

Policy information about availability of computer code

Data collection

Available in the repository:
Data analysis
To review GEO accession GSE191016:
Go to https://www.ncbi.nlm.nih.gov/geo/query/acc.cgi?acc=GSE19l0l6
Enter token shipaqqernanbih into the box

Data analysis

Available in the repository:
Data analysis
To review GEO accession GSE191016:
Go to https://www.ncbi.nlm.nih.gov/geo/query/acc.cgi?acc=GSE19l0l6
Enter token shipaqqernanbih into the box

For manuscripts utilizing custom algorithms or software that are central to the research but not yet described in published literature, software must be made available to editors and reviewers. We strongly encourage code deposition in a community repository (e.g. GitHub). See the Nature Portfolio guidelines for submitting code & software for further information.

March 2021

Data

Policy information about availability of data

All manuscripts must include a data availability statement. This statement should provide the following information, where applicable:

- Accession codes, unique identifiers, or web links for publicly available datasets
- A description of any restrictions on data availability
- For clinical datasets or third party data, please ensure that the statement adheres to our policy

All data generated or analysed during this study are included in this published article (and its supplementary information files).

Field-specific reporting

Please select the one below that is the best fit for your research. If you are not sure, read the appropriate sections before making your selection.

☒ Life sciences ☐ Behavioural & social sciences ☐ Ecological, evolutionary & environmental sciences

For a reference copy of the document with all sections, see nature.com/documents/nr-reporting-summary-flat.pdf

Life sciences study design

All studies must disclose on these points even when the disclosure is negative.

Sample size	In our experiments, a small sample size of each experimental group was used to detect potential effects, for example: a difference of 60 seconds between groups in the Tail Suspension Test in mice, with 80% power. The estimated number of mice was calculated in accordance with the method described in the following book "Biostatistics: A Foundation for Analysis in the Health Sciences, 9th Edition by Wayne W. Daniel. John Wiley & Sons, New York, 2009). This assumes a t-test with a 0.05 significance level. This estimated difference in the immobility-time is based on a previous study by our group (Freitas, A. E. et al. Agmatine, by Improving Neuroplasticity Markers and Inducing Nrf2, Prevents Corticosterone-Induced Depressive-Like Behavior in Mice. Mol Neurobiol 53, 3030-3045, doi:10.1007/s12035-015-9182-6 (2016), in which we observed a 60 seconds difference between the antidepressant treatment and vehicle (control), as well as, a standard deviation of around 30 seconds.
Data exclusions	Mice with optic fiber cannulas displaced were excluded from the analysis.
Replication	Yes, our data were verified regarding reproducibility at least twice and all attempts to reproduce them were successful.
Randomization	Samples were allocated in experimental groups randomly.
Blinding	All data acquisition and analyses were performed by experienced researchers blinded to the experimental conditions.

Reporting for specific materials, systems and methods

We require information from authors about some types of materials, experimental systems and methods used in many studies. Here, indicate whether each material, system or method listed is relevant to your study. If you are not sure if a list item applies to your research, read the appropriate section before selecting a response.

Materials & experimental systems

n/a	Involved in the study	
☐	☒	Antibodies
☐	☒	Eukaryotic cell lines
☒	☐	Palaeontology and archaeology
☐	☒	Animals and other organisms
☒	☐	Human research participants
☒	☐	Clinical data
☒	☐	Dual use research of concern

Methods

n/a	Involved in the study	
☒	☐	ChIP-seq
☒	☐	Flow cytometry
☒	☐	MRI-based neuroimaging

Antibodies

Antibodies used	All antibodies used, including company name and catalog number are provided in the manuscript. Primary antibodies chicken anti-GFP (1:1000; Stock #ab13970; Abcam, Boston, MA), guinea pig anti-Bassoon (presynaptic marker, 1:500; Stock #141004; Synaptic Systems, Goettingen, Germany), goat anti-PSD-95 (postsynaptic marker, 1:250; Stock #ab12093; Abcam, Boston, MA), mouse anti-Iba-1 (microglia marker, 1:500; Stock #MA5-27726; Invitrogen, Waltham, Massachusetts, USA), rabbit IL-1β (proinflammatory cytokine, 1:500; Stock #PA5-105048; Invitrogen, Waltham, Massachusetts, USA), and rabbit anti-NF-κB (transcription factor that regulates inflammatory responses, 1:500; Stock #51-0500; Invitrogen, Waltham, Massachusetts, USA).

Fluorochrome-conjugated secondary antibodies Alexa 488 anti-chicken (1:400; Stock #ab150173; Abcam, Boston, MA), Alexa 647 anti-guinea pig (1:400; Stock #706-605-148; Jackson ImmunoResearch, West Grove, PA), and Alexa 405 anti-goat (1:400; Stock #ab175667; Abcam, Boston, MA).

Validation

All primary antibodies used were validated, as stated on manufacturer websites:

1. Chicken anti-GFP (1:1000; Stock #ab13970; Abcam, Boston, MA): https://www.abcam.com/products/primary-antibodies/gfp-antibody ab13970.html?productWallTab=ShowAll

2. Guinea pig anti-Bassoon (presynaptic marker, 1:500; Stock #141004; Synaptic Systems, Goettingen, Germany): https://www.sysy.com/product/141318

3. Goat anti-PSD-95 (postsynaptic marker, 1:250; Stock #ab12093; Abcam, Boston, MA): https://www.abcam.com/products/primary-antibodies/psd95-antibody-synaptic-marker-ab18258.html?productWallTab=ShowAll

4. Mouse anti-Iba-1 (microglia marker, 1:500; Stock #MA5-27726; Invitrogen, Waltham, Massachusetts, USA): https://www.thermofisher.com/antibody/product/IBA1-Antibody-clone-GT10312-Monoclonal/MA5-27726

5. Rabbit anti-IL-1β (proinflammatory cytokine, 1:500; Stock #PA5-105048; Invitrogen, Waltham, Massachusetts, USA): https://www.thermofisher.com/antibody/product/IL-1-beta-Cleaved-Asp116-Antibody-Polyclonal/PA5-105048

6. Rabbit anti-NF-κB (transcription factor that regulates inflammatory responses, 1:500; Stock #51-0500; Invitrogen, Waltham, Massachusetts, USA): https://www.thermofisher.com/antibody/product/NFkB-p65-Antibody-Polyclonal/51-0500

Eukaryotic cell lines

Policy information about cell lines

Cell line source(s)	????
Authentication	????
Mycoplasma contamination	????
Commonly misidentified lines (See ICLAC register)	????

Animals and other organisms

Policy information about studies involving animals; ARRIVE guidelines recommended for reporting animal research

Laboratory animals

Male, 7 weeks old C57BL/6 (Stock #027; Charles River Labs) and Male and Female 7 weeks CRISPR/Cas9 knockin (Stock #026175; Jackson Laboratory) mice were maintained at constant room with free access to water and food, under a 12:12 h light:dark cycle, 18-23°C temperature and 40-60% air humidity.

No different between sex has been identified.

Wild animals

The study did not involve wild animals.

Field-collected samples

The study did not involve samples collected from the field.

Ethics oversight

The procedures in this study were performed in accordance with the NIH Guide for the Care and Use of Laboratory Animals and approved by the University of California, San Diego (UCSD) Institutional Animal Care and Use Committee

Note that full information on the approval of the study protocol must also be provided in the manuscript.
